# Effectiveness of resistance training in modulating inflammatory biomarkers among Asian patients with sarcopenia: a systematic review and meta-analysis of randomized controlled trials

**DOI:** 10.3389/fimmu.2024.1385902

**Published:** 2024-05-28

**Authors:** Jingxian Xue, Xi Han, Yan Zheng, Qiuxia Zhang, Lingyu Kong

**Affiliations:** ^1^ School of Physical Education, Soochow University, Suzhou, China; ^2^ Sports Business School, Beijing Sport University, Beijing, China

**Keywords:** resistance training, sarcopenia, inflammatory factor, systematic review, meta-analysis

## Abstract

**Objective:**

Given the high incidence of sarcopenia among Asians, it is imperative to identify appropriate intervention methods. The International Clinical Practice Guidelines for Sarcopenia, developed by the International Conference on Sarcopenia and Frailty Research (ICFSR) task force, recommends resistance training (RT) as a primary treatment for managing sarcopenia. Inflammatory biomarkers serve as indicators of sarcopenia. However, there is currently insufficient conclusive evidence regarding the effectiveness of RT in modulating inflammatory biomarker levels among Asian participants with sarcopenia.

**Data sources:**

Four databases were utilized for this study until October 9, 2023. This study focused on randomized controlled trials (RCTs) that examined the effects of RT on interleukin-6 (IL-6), tumor necrosis factor-α (TNF-α), C-reactive protein (CRP), and interleukin-10 (IL-10) about sarcopenia. This study has been registered in the PROSPERO database (CRD42024501855).

**Results:**

The meta-analysis included six studies from Asians involving 278 participants. The results showed a significant decrease in RT for IL-6 (weighted mean difference (WMD) = -0.73, 95% confidence interval (CI) = -1.02 to -0.44; n=5). However, no significant differences were found for TNF-α (WMD = -1.00, 95% CI = -2.47 to 0.46; n=5), CRP (WMD = -0.45, 95% CI = -1.14 to 0.23; n=3), and IL-10 (WMD = 0.13, 95% CI = -3.99 to 4.25; n=2). Subgroup analysis revealed that factors including gender selection, intervention methods, frequency, period, and duration could have a particular effect on the part of inflammatory biomarkers.

**Conclusion:**

RT has been shown to reduce part of the level of inflammatory markers, specifically IL-6, in Asian sarcopenia participants. However, other inflammatory factors, such as TNF-α, CRP, and IL-10, did not show significant changes. Further research should confirm the impact of RT on these indicators and explore the potential effects of various factors on different inflammatory markers, such as diet, body composition, and medications.

**Systematic Review Registration:**

https://www.crd.york.ac.uk/PROSPERO/display_record.php?RecordID=501855, identifier CRD42024501855.

## 1 Introduction

As individuals grow old, the quality and function of their skeletal muscles deteriorate (1). Scientific research indicates that skeletal muscle mass decreases by approximately 1% to 2% every year after age 50, and skeletal muscle strength declines by about 1.5% between 50 and 60 (2). This decline in muscle health may ultimately lead to a condition known as sarcopenia, characterized by a decline in muscle strength among older individuals (3).

Sarcopenia is associated with various negative consequences, including an increased risk of falls, illness, disability, hospitalization, and even mortality (4). Multiple factors contribute to the development of sarcopenia, including dysfunctions in the neuromuscular junction, changes in muscle protein turnover, chronic inflammation, oxidative stress, and unhealthy habits and lifestyles (5). Its mechanism might be associated with changes in microRNA expression, which lead to decreased levels of insulin-like growth factor-1 (IGF-1) and limited signaling in the PI3K/Akt/mTOR pathway, impacting homeostasis and protein synthesis in skeletal muscle cells (6). The disruption of skeletal muscle homeostasis results in increased secretion of inflammatory markers, elevated levels of reactive oxygen species, and activation of nuclear factor κB (NF-κB), ultimately leading to apoptosis and accumulation of oxidative damage, accelerating skeletal muscle loss (7, 8). Sarcopenia is a condition diagnosed with low muscle mass and function (strength or performance), as determined by the consensus of the Asian Working Group for Sarcopenia (AWGS) (9). According to the 2014 AWGS criteria, sarcopenia is a condition that affects between 5.5% to 25.7% of the population; males are more likely to be affected, with a range of 5.1% to 21.0%, compared to females with a range of 4.1% to 16.3% (10). Patients of Asian descent with sarcopenia are at a higher risk of experiencing physical limitation after four years, delayed mobility after seven years, and death after ten years (11).

Given the high incidence of sarcopenia among Asians, it is imperative to identify appropriate intervention methods. The International Clinical Practice Guidelines for Sarcopenia, developed by the task force of the International Conference on Sarcopenia and Frailty Research (ICFSR), strongly recommend incorporating resistance training (RT) into routines to enhance muscle strength and physical performance (12, 13). RT induces muscle contraction by utilizing various forms of external resistance, such as kettlebells, dumbbells, elastic bands, and self-weight. One primary protective mechanism of RT for muscle is it can boost the synthesis of muscle proteins through the upregulation of IGF-1 expression, which is crucial for muscle synthesis but is often deficient in sarcopenia patients; it also enhances skeletal muscle mass and function via the PI3K/Akt/mTOR pathway and improves the activation, proliferation, and differentiation of aged skeletal muscle satellite cells, promoting the rise in cell amounts to enhances skeletal muscle protein synthesis and stimulates muscle hypertrophy (14, 15). RT is considered safe and has been shown to effectively reduce chronic low-grade inflammation in senior citizens (16). In 2018, Johanna et al. demonstrated that a combined aerobic and RT program lasting 24 weeks can reduce inflammation associated with reductions in abdominal fat mass (17). It demonstrates the great potential of RT in alleviating sarcopenia.

This persistent low-grade inflammation, known as Inflammaging, is closely related to aging. Inflammaging is characterized by higher levels of serum inflammatory markers, such as interleukin-6 (IL-6), tumor necrosis factor-alpha (TNF-α), and c-reactive protein (CRP) (18). The upregulated levels of these factors are closely related to the activation of NF-κB, which accelerates the development of inflammation. Briefly, TNF-α activates the Inhibitor of κB kinase by binding to cell membrane receptors, leading to the degradation of the Inhibitor of NF-κB, subsequent release of NF-κB, and induction of transcription of target genes, which ultimately results in the activation of NF-κB and transcription of inflammatory cytokine genes (19, 20). As an acute-phase reactant, CRP can also bind to its receptor CD32/CD64, activating the NF-κB signaling pathway to induce inflammation, leading to a decline in muscle strength (21, 22). A previous meta-analysis revealed that higher inflammatory markers, including IL-6, TNF-α, and CRP, were significantly linked to lower skeletal muscle strength and muscle mass (23). Enhancing the expression/level of these inflammatory factors could contribute to decreased skeletal muscle strength and muscle mass (24). Furthermore, anti-inflammatory cytokines such as interleukin-10 (IL-10) inhibit mTORC1 activation through STAT3 (25). It can inhibit the expression and function of pro-inflammatory factors, thus slowing down muscle atrophy and delaying the onset of sarcopenia (26). Therefore, inflammatory factors such as IL-6, TNF-α, CRP, and IL-10 play a role in the development of sarcopenia and could serve as markers to evaluate the severity of sarcopenia (27–29).

Existing evidence-based medicine meta-analyses have shown that RT significantly positively affects grip strength, gait speed, and the skeletal muscle index in participants with sarcopenia. However, management approaches for sarcopenia tend to concentrate on the clinical aspects of the condition, overlooking the importance of addressing the underlying biological changes that occur before symptoms become apparent (30). Effective sarcopenia interventions should target participants’ molecular and cellular changes (31). Despite this, there is still no consensus on the efficacy of RT in treating sarcopenia on inflammatory cytokines at the molecular level. In order to make our argument about the effect of RT on inflammatory factors possible, many scientists have conducted high-quality experimental studies in recent years. In 2018, Chen et al. (32) demonstrated that an eight-week program of kettlebell exercises decreased CRP levels in older women with sarcopenia, but there were no significant differences in IL-6 or TNF-α levels. In 2024, Heo et al. (33) also reported a positive effect of a 12-week RT on factors associated with sarcopenia in older participants, such as muscle mass and inflammatory cytokines. These studies have provided the foundation for our research. We hypothesized that RT could suppress the expression of pro-inflammatory markers, such as IL-6, TNF-α, and CRP, while promoting the expression of anti-inflammatory factors. Therefore, this systematic review and meta-analysis aim to provide conclusive evidence on the effect of RT on inflammatory markers in Asian participants with sarcopenia from an evidence-based medicine perspective. Additionally, this study aims to identify specific forms of RT that effectively modulate inflammatory markers in these participants.

## 2 Methods

This assessment adheres to the PRISMA (Preferred Reporting Items for Systematic Reviews and Meta-Analyses) guidelines and has been duly recorded in the International Prospective Registry of Systematic Reviews (PROSPERO: CRD42024501855).

### 2.1 Literature search

Four databases, namely PubMed, Web of Science, Embase, and Cochrane Library, were utilized for this study. The search formula was created by combining terms related to sarcopenia, resistance training, and inflammatory biomarkers. The search included publications without language or country restrictions until October 9, 2023. The search strategy combined the following medical subject headings with accessible terms and matching synonyms: ‘sarcopenia,’ ‘sarcopenias,’ ‘resistance training,’ ‘resistance exercise,’ ‘strength training,’ ‘strength exercise,’ ‘weight lifting strengthening program,’ ‘weight lifting exercise program,’ ‘weight bearing strengthening program,’ ‘weight-bearing exercise program,’ ‘inflammation,’ ‘innate inflammatory response,’ ‘c reactive protein,’ ‘CRP,’ ‘interleukin 6,’ ‘IL-6,’ ‘interleukin 10,’ ‘IL-10,’ ‘tumor necrosis factor-alpha,’ ‘TNF-α.’ The complete search strategy for each database is available in the **Supplementary Material**.

### 2.2 Eligibility criteria

The inclusion criteria for this study were as per the PICOS protocol:

1. The participants (P) were Asian adults diagnosed with sarcopenia;
2. The interventions (I) included, but were not limited to, various forms of RT, such as kettlebell training, elastic band exercises, exercises with elastic balls, and self-weight exercises;
3. The comparison/control (C) group received non-RT interventions such as usual care, routine care, routine activities, and health education;
4. The outcomes (O) measured included at least one of the inflammatory markers: IL-6, TNF-α, CRP, and IL-10;
5. This study design (S) only involved randomized controlled trials (RCTs).

Furthermore, this study excluded the subsequent studies:

1. The study designs were not RCTs;
2. The interventions or controls were deemed irrelevant;
3. The participants were not sarcopenic or of Asian descent;
4. The outcome indicators of inflammatory markers do not provide detailed data.

### 2.3 Study selection and data extraction

The literature screening was conducted using the Endnote citation management software (EndNote X9.3.3). Information extracted from the literature included the first author, year of publication, country/region, sample size, participant characteristics (gender, age, sarcopenia diagnostics), intervention elements (methods, duration, frequency, and period), control group (CG), and outcomes. The mean and standard deviation of the changes before and after intervention in both groups will be directly extracted or indirectly calculated using the sample size, median, range, or interquartile range (34–36). Two researchers (X.J. and K.L.) independently conducted the literature screening and data extraction. They consulted each other after completion and referred any disagreements to a third researcher (Z.Q.) for further discussion and decision.

### 2.4 Methodological quality

In order to evaluate the risk of bias in the RCTs included in our study, we utilized the Cochrane Risk-of-Bias Tool for Randomized Interventions (RoB). This tool evaluates the risk of bias in six domains: random sequence generation, allocation concealment, blinding of participants and personnel, blinding of outcome assessment, incomplete outcome data, and selective outcome reporting. Each domain was categorized as ‘low risk of bias,’ ‘some concerns,’ or ‘high risk of bias.’ Additionally, we employed the Grades of Recommendation, Assessment, Development, and Evaluation (GRADE) approach to determine the certainty of evidence. The GRADE approach considers factors such as study design, consistency of results, directness of evidence, precision, and publication bias to assess the quality of evidence and assign a grade of strength. Utilizing the RoB tool and the GRADE approach, we thoroughly evaluated the risk of bias and certainty of evidence in the RCTs included in this study, ensuring a robust assessment of the research findings. In cases where there were disagreements between the initial reviewers (X.J. and K.L.), a third researcher (Z.Q.) was involved to resolve them.

### 2.5 Statistical analysis

We extracted the sample size for each study and assessed the difference in data between the experimental group and the CG before and after the intervention. This assessment included the average and standard deviation of inflammatory biomarkers to evaluate the effect of RT on the regulation of inflammatory markers in sarcopenia. We calculated the weighted mean difference (WMD) and 95% confidence interval (CI) for continuous outcomes. All outcome data were converted to a standardized unit before calculating the WMD. The units of IL-6, TNF-α, and IL-10 are pg/ml, while the unit for CRP is mg/l. We also calculated the I² statistic and 95% CI to assess heterogeneity. If the heterogeneity exceeded 50%, we used the random effects model; if the heterogeneity was below 50%, we utilized the fixed effects model. Heterogeneity was considered significant if the I² value was above 50% or the *P* value was below the predefined significance level (*P* = 0.05). Conversely, if the I² value was 50% or less and *P* ≥ 0.05, we did not consider the heterogeneity significant. The I² value less than 50% indicates low heterogeneity, and the I² value greater than 50% indicates high heterogeneity. *P* of effect size less than 0.05 was considered statistically significant. We conducted a sensitivity analysis by excluding each included study to assess the robustness of the combined results. We also used Begg’s regression asymmetry test and Egger’s regression plot test to assess the risk of publication bias. Additionally, we performed subgroup analysis to explore the source of heterogeneity and potential factors that may influence the results, such as gender, intervention methods, intervention period, intervention frequency, and intervention duration. All statistical analyses were conducted using Stata SE version 15.

## 3 Results

### 3.1 Literature search

**Figure 1:**
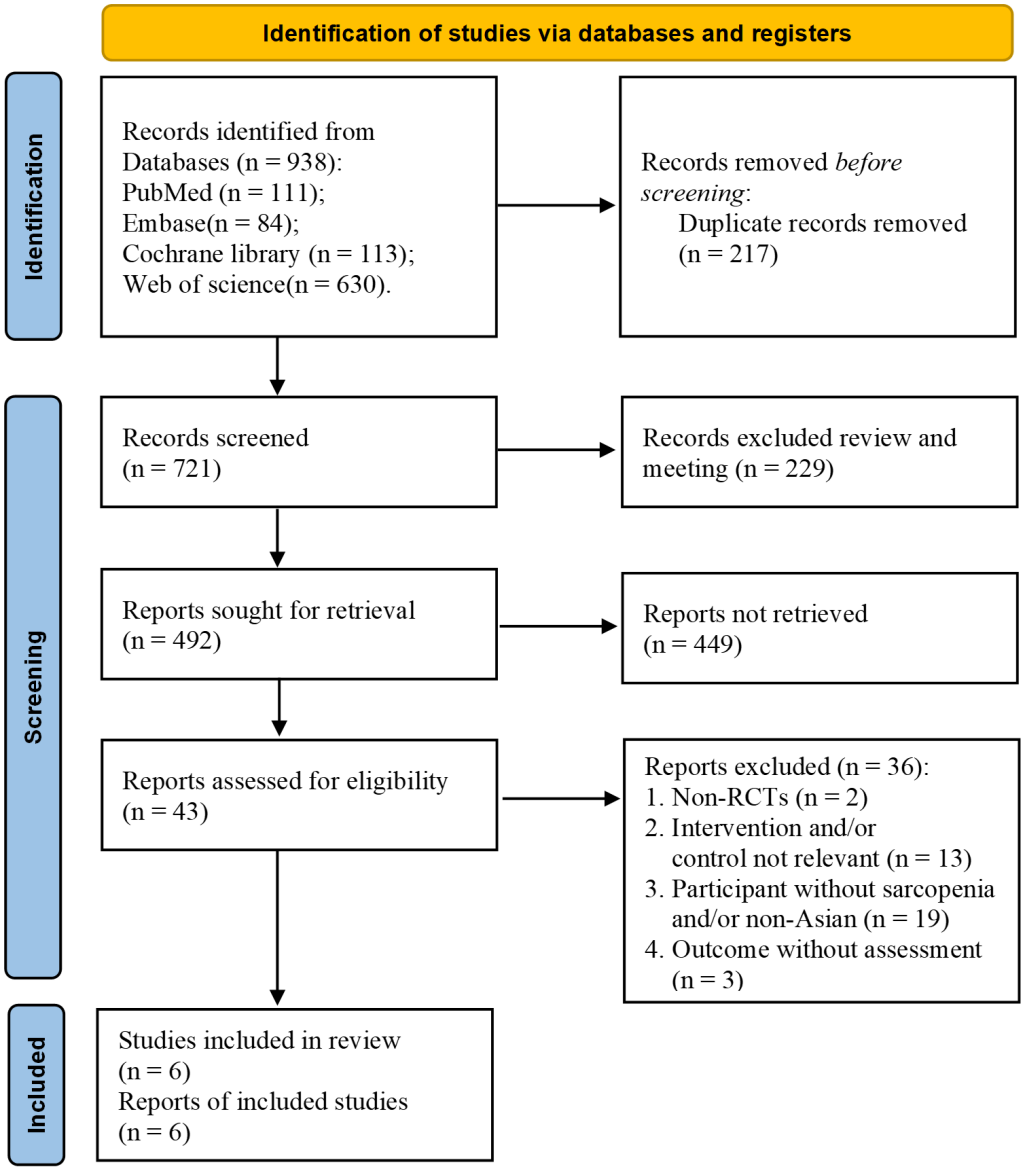
Flow diagram of this study selection process.

After conducting a database search, 938 studies were found. Following the removal of duplicate studies, 721 studies remained. Among them, 43 studies were found to be eligible for inclusion based on their titles and abstracts. After a full-text review, only six studies met the inclusion criteria (**Figure 1**).

### 3.2 Study characteristics

Between 2016 and 2023, six RCTs of parallel design were conducted in Asia, specifically in China, Japan, Korea, Chinese Taipei, and Thailand (**Table 1**).

**Table 1:** Characteristics of studies included in systematic reviews.

Study	Country/region	Sample size (RT/CG)	Gender (M/F)	Age	Sarcopenia diagnostics	Characteristics of the intervention	CG	Outcome
(32)	Chinese Taipei	17/16	RT: 0/17 CG: 0/16	RT: 66.7 ± 5.3 CG: 68.3 ± 2.8	ASMI by BIA,F: < 5.7 kg/m^2^; Grip strength,F: < 18 kg.	Kettlebell training protocol: Time: 60 mins, 2 sessions/week, 8 weeks; 60% - 70% of 1 RM; 3sets, 8 - 12 reps and 11 movements.	No exercise training program	CRP IL-6 TNF-α
(37)	China	21/20	RT: 9/12 CG: 12/8	56.80 ± 19.21	SMI by BIA, M: < 7.0 kg/m^2^, F:< 5.7 kg/m^2^; Grip strength, M: < 26 kg, F: < 18 kg; Gait speed < 0.8 m/s.	RT protocol: Time: 3 sessions/week, 12 weeks; (self-weight and elastic balls).	Routine care	CRP IL-6 IL-10 TNF-α
(33)	Korea	20/20	RT: 9/11 CG: 10/10	RT: 67.45 ± 4.70 CG: 67.95 ± 5.05	ASMI by DXA, M: < 7.0 kg/m^2^, F: < 5.7 kg/m^2^; Grip strength, M: < 26 kg, F: < 18 kg.	Strengthening exercise programs protocol: Time: 50 mins, 3 sessions/week, 12 weeks; 1^st^ month of 10 RM, 2^nd^ month of 9 RM, 3^rd^ month of 8 RM (instruments).	Meditation or light stretching and no exercise training program	IL-6 IL-10 TNF-α
(38)	Japan	35/34	RT: 0/35 CG: 0/34	RT: 81.4 ± 4.3 CG: 81.1 ± 5.1	SMI by DXA, F < 5.7 kg/m^2^; Grip strength, F < 18 kg; Gait speed < 1.0 m/s.	Resistance and weight-bearing exercise: Time: 3 sessions/week, 12 weeks; 3sets of 10 reps (self-weight and resistance band).	Health education	IL-6 CRP
(39)	China	20/14	RT: 7/13 CG: 5/9	69.95 ± 4.72	ASMI by DXA; KES, M: ≤ 18 kg, F: ≤ 16 kg; Gait speed ≤ 0.8m/s.	Physical exercise protocol: RT integrated with functional tasks and balance: Time: 90 mins,2 sessions/week, 24 weeks;	Standard care	CRP TNF-α
(40)	Thailand	28/29	RT: 10/20 CG: 6/24	≥ 60	LMM by BIA, M: < 7.0 kg/m^2^; F: < 5.7 kg/m^2^; Grip strength, M: < 28 kg, F: < 18 kg.	RT protocol: Time: 2 sessions/week, 12 weeks; 3 sets, 10 mins/set and 10 movements (elastic band).	Routine activities	IL-6 TNF-α

RT, Resistance training; CG, Control group; BIA, Bioelectrical impedance analysis; DXA, Dual-energy X-ray absorptiometry; M, Male; F, Female; ASMI, Appendicular skeletal muscle index; SMI, Skeletal muscle mass index; KES, Knee extension strength; AMI, Appendicular mass index; LMM, Low muscle mass; Mins, Minutes; Rm, Repetition maximum; Reps, Repetitions; RT, Resistance training; TNF-α, Tumor necrosis factor-alpha; IL-6, Interleukin-6; IL-10, Interleukin-10; CRP, C reactive protein.

### 3.3 Participants

The present study included a total of 278 individuals diagnosed with sarcopenia, with ages ranging from 56 to 81 years old. These individuals were part of six distinct research papers. Among the participants, 145 were randomly assigned to the RT intervention group, while the remaining 133 were allocated to the CG. Two studies focused exclusively on female participants (32, 38), while the other four included individuals of both genders in their samples (33, 37, 39, 40). Grip strength, skeletal muscle mass, and gait speed are commonly used to identify and characterize sarcopenia. Grip strength is employed to assess the likelihood of sarcopenia, skeletal muscle mass is used to confirm its presence, and gait speed is utilized to evaluate the severity of the condition (10, 41). The diagnosis of sarcopenia is determined by assessing muscle mass, which can be evaluated through Bioelectrical Impedance Analysis (BIA) or dual-energy X-ray absorptiometry (DXA). Three studies reviewed used BIA (32, 37, 40), and the other three used DXA to evaluate skeletal muscle mass (33, 38, 39) (**Table 1**).

### 3.4 Intervention

This study included six studies that examined various RT regimens for different intervention groups. The regimens consisted of kettlebell exercises, elastic band exercises, instruments, and mixed styles and were scheduled 2 to 3 times per week for an intervention period of 8 to 24 weeks. Three of these studies did not mention the duration of a single training session (37, 38, 40), while the remaining sessions lasted between 50 and 90 minutes. In four studies, participants in the CG received usual care and advice through a medical visit or a telephone call (32, 37–39). In two studies, advice on physical activity was provided (33, 40) (**Table 1**).

### 3.5 Outcomes

Among the six studies included, five evaluated IL-6 levels (32, 33, 37, 38, 40), five measured TNF-α levels (32, 33, 37, 39, 40), four studies assessed CRP levels (32, 37–39), and two reported IL-10 levels (33, 37). Furthermore, we observed a significant imbalance in our results during the initial analysis, which we attributed to one study by Dong et al. (37) reporting a mean CRP value at least 1000 times lower than the mean of the other studies (32, 38, 39). Thus, based on previous studies of the same type (42), only three studies assessing CRP levels were included (32, 38, 39). Additionally, five of the six included studies reported the concentrations of inflammatory markers before and after the intervention (32, 33, 37–39). Only one study provided information on the change in inflammatory marker concentration (40) (**Table 1**).

### 3.6 Methodological quality

**Figure 2:**
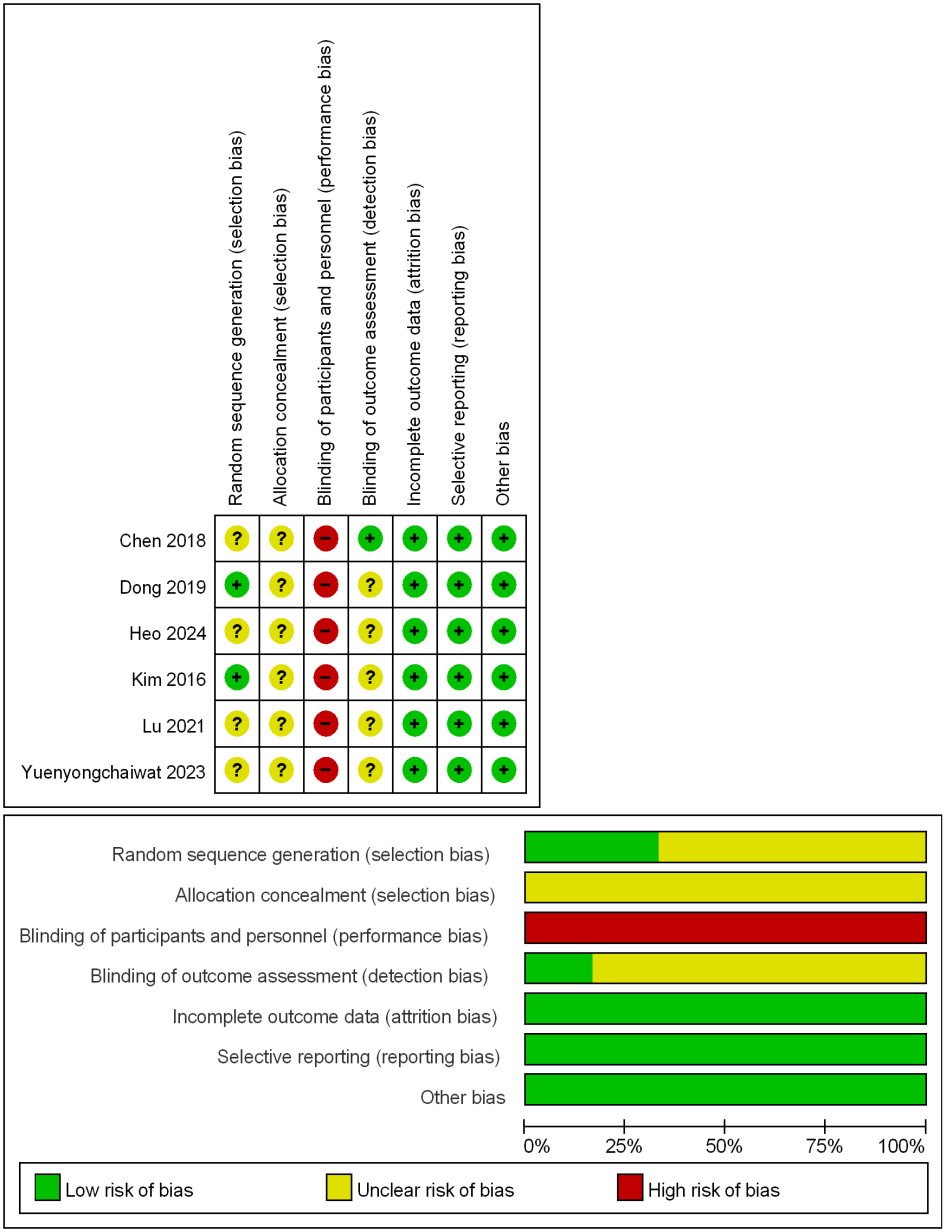
Methodological quality assessment of the included RCTs using the RoB tool.

Among the analyzed studies, four exhibited low bias in generating the sequence (32, 33, 39, 40). However, the two studies could not have mentioned specific measures for generating the sequence, raising concerns about their methodology (37, 38). Regarding hidden allocation, none of the six studies provided enough information about hidden assignments, which casts doubt on bias. The blinding of participants and personnel involves withholding information about the treatment provided, and all six studies presented a high risk of bias due to the resistance training, introducing uncertainty and potential bias. Only one assessment of inflammatory markers, which involved recruiting qualified healthcare workers, was considered low risk in terms of blinding of outcome assessment (32). Conversely, the remaining five studies failed to provide detailed information on the blind outcome assessment method, raising concerns (33, 37–40). Finally, all six studies demonstrated low risk regarding incomplete outcome data, selective reporting, and other biases (**Figure 2**).

### 3.7 Meta-analyses

The effects of RT on TNF-α (I^2 ^= 88.2%, *P* < 0.001) and IL-10 (I^2 ^= 80.6%, *P* = 0.023) exhibited a significant level of heterogeneity. However, the effects of RT on IL-6 (I^2 ^= 21.5%, *P* = 0.278) and CRP (I^2 ^= 0.0%, *P* = 0.604) demonstrated consistent results across studies with no significant heterogeneity.

**Figure 3:**
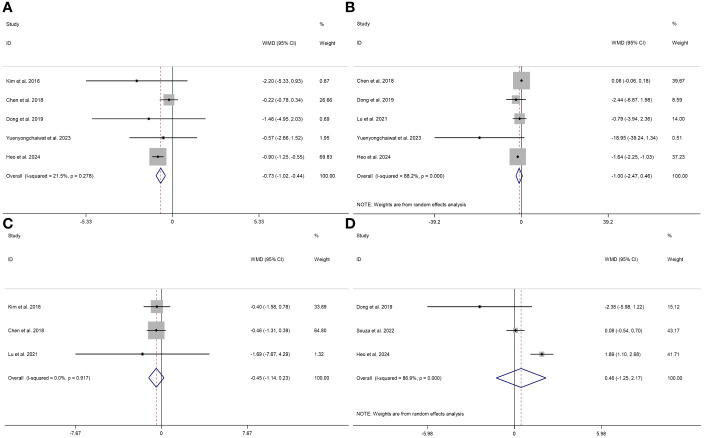
Forest plot describing the effect of RT on the levels of IL-6 **(A)**, TNF-α **(B)**, CRP **(C)**, and IL-10 **(D)** in sarcopenia. Weighted means difference was used to analyze the data, with a 95% confidence interval used as effect sizes. The fixed-effects model was applied to IL-6 **(A)** and CRP **(C)**, while the random-effects model was used for TNF-α **(B)** and IL-10 **(D)**.

This study used a fixed effects model to analyze the IL-6 and CRP indicators, while a random effects model for the TNF-α and IL-10 indicators. When combining the effect size, the results demonstrated a statistically significant difference and low heterogeneity in the effect of RT on IL-6 (WMD = -0.73 pg/ml, 95% CI = -1.02 to *-*0.44, *P* < 0.001, I^2 ^= 21.5%); no statistical difference and high heterogeneity in the effect of RT on TNF-α (WMD = -1.00 pg/ml, 95% CI = -2.47 to 0.46, *P* = 0.141, I^2 ^= 88.2%; no statistical difference and low heterogeneity in the effect of RT on CRP (WMD = *-*0.45 mg/l, 95% CI = -1.14 to 0.23, *P* = 0.194, I^2 ^= 0.0%); no statistical difference and high heterogeneity in the effect of RT on IL-10 (WMD = 0.13 pg/ml, 95% CI = -3.99 to 4.25, *P* = 0.951, I^2 ^= 80.6%). These results suggest that RT improves IL-6 but not TNF-α, CRP, and IL-10 in participants with sarcopenia (**Figure 3**).

### 3.8 Publication bias

Begg’s and Egger’s tests were used to analyze the publication bias in this study. The Begg regression asymmetry test and Egger’s regression plot indicated no significant differences in TNF-α (Begg’s test, *P* = 0.462; Egger’s test, *P* = 0.186), IL-6 (Begg’s test, *P* = 1; Egger’s test, *P* = 0.851), and CRP (Begg’s test, *P* = 0.602; Egger’s test, *P* = 0.286). These findings show the trustworthiness of the results obtained from this meta-analysis (**Supplementary Material**).

### 3.9 Sensitivity analysis

**Figure 4:**
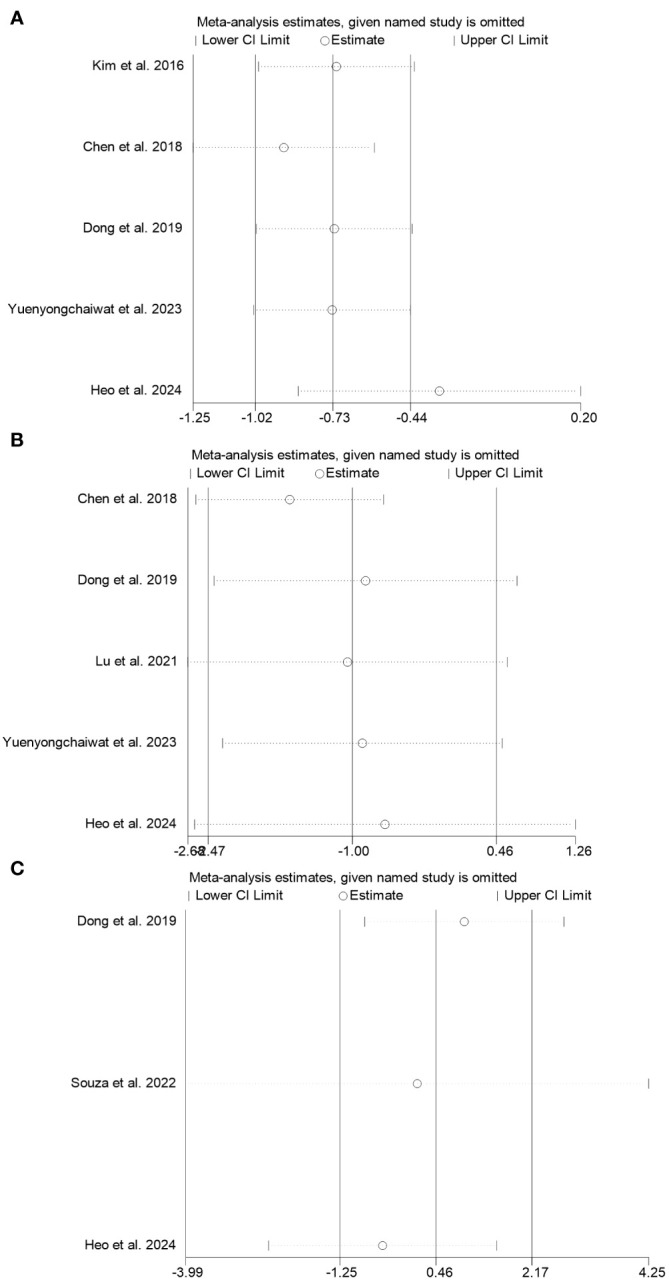
Sensitivity analysis was performed on the effects of RT on TNF-α **(A)**, IL-6 **(B)**, and CRP **(C)** in sarcopenia.

We conducted a sensitivity analysis to evaluate the stability and reliability of the result. In this analysis, we systematically excluded each study to assess the potential effect on the findings. Due to the inclusion of only two studies on IL-10, conducting a sensitivity analysis was impossible. The findings reveal that removing any single study did not yield robust results for TNF-α and IL-6, while robust results were observed for CRP, as shown in **Figure 4**.

### 3.10 Result of subgroup analysis

Several confounders, including gender, intervention methods, period, frequency, and duration, were performed by stratified subgroup analyses to identify the sources of heterogeneity and intervention parameters. In addition, there was no statistical difference in the overall effect size between CRP and IL-10, and CRP was of low heterogeneity, with only two studies on IL-10. Hence, this subgroup analysis focused solely on IL-6 and TNF-α. The analysis revealed a significant effect on the levels of IL-6 and TNF-α under the specific factors (**Table 2**).

**Table 2:** Subgroup analysis of the effects of RT on TNF-α (A) and IL-6 (B) in sarcopenia.

(A) IL-6							
Variate	Group	Heterogeneity		Mean Difference, 95% CI	Statistical *P*	Literature quantity	Sample
		*P*	I^2^				
Gender	Female	0.222	33.0%	-0.28 (-0.84, 0.27)	0.319	2	101
	Gender mixed	0.907	0.0%	-0.90 (-1.24, -0.55)	<0.001	3	138
Intervention methods	Unitary type	0.131	50.7%	-0.71 (-1.00,-0.42)	<0.001	3	130
	Mixed type	0.751	0.0%	-1.87(-4.20, 0.46)	0.115	2	109
Intervention frequency	< 3 sessions	0.751	0.0%	-0.24 (-0.79, 0.30)	0.380	2	90
	≥ 3 sessions	0.688	0.0%	-0.92 (-1.27, -0.58)	<0.001	3	149
Intervention period	< 12 weeks	–	–	0.06 (-0.06, 0.18)	0.310	1	33
	≥ 12 weeks	0.836	0.0%	-0.91 (-1.25, -0.57)	<0.001	4	206
Intervention duration	≤ 60 minutes	0.086	59.2%	-0.73 (-1.02, -0.43)	<0.001	3	141
	Duration unknown	0.668	0.0%	-0.80(-2.60, 0.99)	0.379	2	98
Overall		0.278	21.5%	-0.73 (-1.02, -0.44)	<0.001	5	239
(B) TNF-α							
Variate	Group	Heterogeneity		Mean Difference, 95% CI	Statistical *P*	Literature quantity	Sample
		*P*	I^2^				
Gender	Female	–	–	0.06 (-0.06, 0.18)	0.310	1	33
	Genders mixed	0.362	6.3%	-1.64 (-2.60, -0.46)	0.001	4	177
Intervention methods	Unitary type	<0.001	93.8%	-0.89 (-2.62, 0.83)	0.309	3	130
	Mixed type	0.550	0.0%	-1.35 (-3.91, 1.22)	0.303	2	80
Intervention frequency	< 3 sessions	0.161	45.2%	-0.45 (-2.78, 1.89)	0.708	3	129
	≥ 3 sessions	0.724	0.0%	-1.65 (-2.26, -1.05)	<0.001	2	81
Intervention period	< 12 weeks	–	–	0.06 (-0.06, 0.18)	0.310	1	33
	≥ 12 weeks	0.362	6.3%	-1.64 (-2.60, -0.46)	0.001	4	177
Intervention duration	> 60 minutes	–	–	-0.79(-3.94, 2.36)	0.623	1	34
	≤ 60 minutes	<0.001	96.6%	-0.76(-2.43, 0.90)	0.320	2	79
	Duration unknown	0.119	58.8%	-7.60(-22.60, 7.39)	0.369	2	98
Overall		<0.001	88.2%	-1.00 (-2.47, 0.46)	0.141	5	210

CI, confidence interval; BIA, bioelectrical impedance analysis; DXA, dual-energy X-ray absorptiometry.

#### 3.10.1 Gender

When comparing subgroups according to gender, we observed significant differences with RT on IL-6 and TNF-α levels in participants with sarcopenia (*P* < 0.01 for both genders mixed). In the genders mixed group, there is a significant decrease in IL-6 (N = 3, WMD = -0.90, 95% CI = -1.24 to -0.55, *P* < 0.001, I^2 ^= 0.0%) and TNF-α (N = 4, WMD = -1.64, 95% CI = -2.60 to -0.46, *P* = 0.001, I^2 ^= 6.3%), whereas there are no significant changes in TNF-α and IL-6 in the female.

#### 3.10.2 Intervention methods

When comparing subgroups according to the intervention methods, we only observed significant differences with RT on IL-6 levels in participants with sarcopenia (*P* < 0.01 for unitary type). In the unitary type group, there is a significant decrease in IL-6 (N = 3, WMD = -0.71, 95% CI = -1.00 to -0.42, *P* < 0.001, I^2^ = 50.7%), whereas there are no significant changes in IL-6 with mixed type and TNF-α.

#### 3.10.3 Intervention frequency

When comparing subgroups according to the intervention frequency, we observed significant differences with RT on IL-6 and TNF-α levels in participants with sarcopenia (*P* < 0.01 for both at least three times per week). In the more than three times per week group, there is a significant decrease in IL-6 (N = 3, WMD = *-*0.92, 95% CI = *-*1.26 to *-*0.57, *P* < 0.001, I^2 ^= 0.0%) and TNF-α (N = 4, WMD = -1.64, 95% CI = -2.60 to -0.46, *P* = 0.001, I^2^ = 6.3%), whereas there are no significant changes in TNF-α and IL-6 with less than three times per week.

#### 3.10.4 Intervention period

When comparing subgroups according to the intervention period, we observed significant differences with RT on IL-6 and TNF-α levels in participants with sarcopenia (*P* < 0.01 for both at least 12 weeks). In the equal or more than 12 weeks group, there is a significant decrease in IL-6(N = 3, WMD = *-*0.91, 95% CI = *-*1.25 to *-*0.57, *P* < 0.001, I^2^ = 0.0%) and TNF-α (N = 4, WMD = *-*1.64, 95% CI = *-*2.60 to *-*0.46, *P* = 0.001, I^2^ = 6.3%), whereas there are no significant changes in TNF-α and IL-6 with less than 12 weeks.

#### 3.10.5 Intervention duration

When comparing subgroups according to the intervention duration, we only observed significant differences with RT on IL-6 levels in participants with sarcopenia (*P* < 0.01 for 60 minutes or less). In the 60 minutes or less group, there is a significant decrease in IL-6 (N = 3, WMD = *-*0.91, 95% CI = *-*1.25 to *-*0.57, *P* < 0.001, I^2^ = 59.2%), whereas there are no significant changes in IL-6 with more than 60 minutes and TNF-α.

### 3.11 The overall quality of the evidence

The assessments of the six studies according to GRADE are provided in **Supplementary Material**, with the quality of evidence for IL-6 as moderate, CRP and TNF-α as low, and IL-10 as very low (**Supplementary Material**).

## 4 Discussion

**Figure 5:**
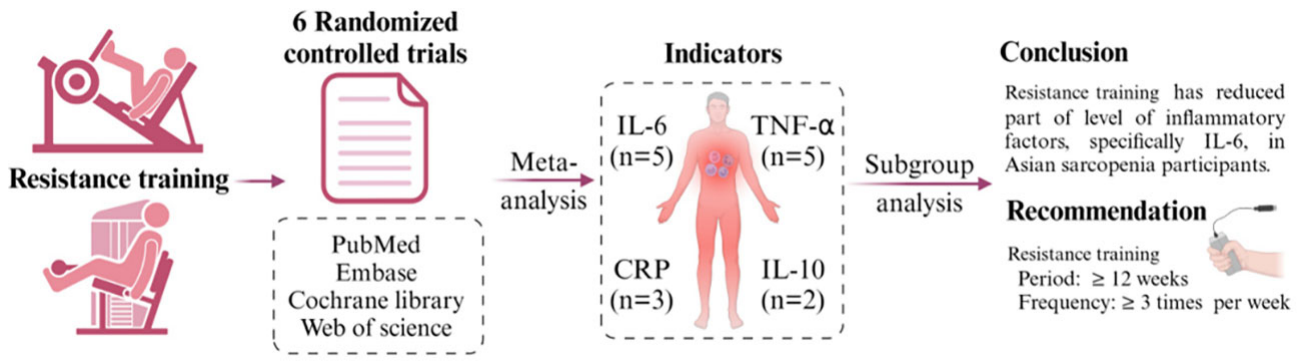
The summary of the meta-analysis.

In this systematic analysis and meta-analysis, this study explores the effect of RT compared to conventional interventions in Asian participants with sarcopenia, focusing on its potential to reduce inflammatory factors. Our study’s results indicate that RT significantly reduces part of inflammatory markers among participants with sarcopenia, specifically IL-6 levels, while no significant changes in TNF-α, CRP, and IL-10 levels (**Figure 5**).

### 4.1 Effect of RT on IL-6 and TNF-α

IL-6, a crucial cytokine involved in the immune response, tissue regeneration, and metabolism, is secreted by various cell types, including monocytes, T lymphocytes, fibroblasts, and endothelial cells (43). In cases of infection or injury, production of IL-6 increases rapidly to boost immune and repair processes. However, excessive production of IL-6 and disruptions in its receptor signaling pathways can contribute to aging-related diseases (44). The elevation in IL-6 levels, which occurs with age, is a significant factor in the decline of skeletal muscle strength, quality, function, and training-mediated adaptation (45); TNF is a cytokine linked to chronic inflammatory and metabolic disorders, which can result in decreased protein, fat, and glycogen synthesis in skeletal muscle, leading to reduced muscle mass and strength (46). TNF-α production could be affected by various factors, including physical activity, diet, smoking, and age. The formation of reactive oxygen species is associated with this marker (47). The meta-analysis of 22 studies involving post-menopausal women and 14 studies in community-dwelling older adults with type 2 diabetes demonstrated that exercise training significantly decreased IL-6 levels (48, 49). In the meta-analysis conducted on elderly individuals, the effect of exercise on inflammatory markers revealed a reduction in IL-6 levels but no effect on TNF-α (50). Another meta-analysis on elderly individuals investigated the potential of RT to decrease IL-6 levels without altering TNF-α. (51). Our results support their findings that a significant decrease in IL-6 levels with RT for sarcopenia, with no significant change in TNF-α levels. Nevertheless, TNF-α did exhibit a significant change under certain specific factors.

Subgroup analysis was conducted by considering factors. The analysis revealed a significant effect of these specific factors on the levels of IL-6 and TNF-α. This finding aligns with the recommendations by intervention factor of the meta-analysis on the rehabilitation of elderly patients with sarcopenia (52). Regarding IL-6 and TNF-α, the genders mixed group showed a more positive effect than the pure-female group. An experiment revealed a correlation between low serum myostatin levels and low skeletal muscle mass in men, but this association was not in women (53). Therefore, subgroup analysis regarding the gender of this discrepancy in the findings could be linked with gender-specific characteristics of women.

Moreover, subgroup analysis further revealed that RT exhibits higher effectiveness when compared to conventional intervention performed at least three times a week. The International Exercise Recommendations in Older Adults (ICFSR) recommends RT thrice weekly to promote muscle mass and performance in the older (54); RT should be performed as a long-term intervention lasting for a minimum of 12 weeks to achieve optimal effectiveness. A meta-analysis examining the relationship between RT dosage and muscle strength in older adults suggests that engaging in RT for a sufficiently long training cycle can enhance muscle strength (55). At the same time, only IL-6 showed a significant change with unitary intervention types and duration equal to or less than 60 minutes, while TNF-α showed no change. Therefore, the intervention period for RT should be at least 12 weeks, with a frequency of at least three times per week.

### 4.2 Effect of RT on CRP

In addition to IL-6 and TNF-α, CRP plays a critical role in sarcopenia. CRP is a clinical inflammation marker (56). When inflammation or tissue damage occurs, the liver produces CRP in response to IL-6, increasing the blood’s CRP concentration (57). In 2018, Tomeleri et al. (58) found that the 12-week RT program had a beneficial effect on reducing metabolic syndrome and CRP levels in older women. According to the meta-analysis, physical exercise can significantly reduce CRP levels in older individuals, regardless of the type of physical activity (48, 49, 59). Another meta-analysis focusing on patients with type 2 diabetes mellitus demonstrated that exercise effectively reduces CRP levels (60). Our findings contradict previous studies regarding CRP, possibly due to the difference in the number of CRP studies considered. The meta-analysis above included more than 10 CRP studies, whereas our study focused on only three. Consequently, it is imperative to conduct research that incorporates a more significant number of studies to comprehensively examine the effectiveness of RT on CRP levels in Asian participants with sarcopenia.

### 4.3 Effect of RT on IL-10

On the other hand, IL-10 is a cytokine known for its anti-inflammatory properties. It can hinder the activity of monocytes and macrophages and reduce the production of pro-inflammatory cytokines (61). In 2022, de Sá Souza et al. (62) found no significant difference in IL-10 and TNF-α levels among older individuals with sarcopenia who underwent RT. A meta-analysis reported that RT did not affect IL-10 levels in patients with type 2 diabetes (63). Our study’s findings align with previous findings that IL-10 levels were not significantly affected by RT. IL-10 focuses on only two studies; it is essential to consider that the sample size is too small to reflect the authenticity of IL-10 results. Consequently, it is imperative to conduct research that incorporates a more significant number of studies to comprehensively examine the effectiveness of RT on IL-10 levels in Asian participants with sarcopenia.

### 4.4 Strength and limitations

This study presents preliminary evidence of the effectiveness of RT in reducing inflammatory factors in participants with sarcopenia. We specifically focused on sarcopenia and conducted subgroup analyses based on participant gender and the type of RT intervention. Based on these results, our study highlights in which circumstances RT is most effective, which is a significant strength of our study.

There are still some limitations to interpreting the results. Some studies needed more information for meta-analysis, and most studies have room for improvement in random assignment concealment and double-blinding. Moreover, the included studies exhibit apparent heterogeneity, and the sensitivity analysis lacks robustness, which may affect the accuracy of the pooled results. In addition, the limited number of included studies restricts the ability to draw definitive conclusions. Although subgroup analyses identify potential sources, more detailed information on training intensity and detailed age of participants with sarcopenia is needed. Therefore, the meta-analysis results should be interpreted cautiously because of the lack of robustness of the sensitivity analysis and high heterogeneity. Future high-quality studies will be necessary for verification.

## 5 Conclusions

In conclusion, this study found that compared to conventional interventions, RT has reduced IL-6 levels among Asian patients with sarcopenia. They had a relatively inconspicuous ability to regulate other inflammatory factors such as TNF-α, CRP, and IL-10.

Based on the available results, this study recommends that if we want to reduce inflammatory factors in participants with sarcopenia effectively, RT should last at least 12 weeks with a frequency of at least three sessions per week to reduce IL-6 levels in participants with sarcopenia. Future research is needed to confirm the effects of RT on these indicators and investigate the potential effects of various factors, including diet, body composition, and medications, on different inflammatory markers.

## Data availability statement

The raw data supporting the conclusions of this article will be made available by the authors, without undue reservation.

## Author contributions

JX: Conceptualization, Data curation, Formal analysis, Methodology, Software, Visualization, Writing – original draft. XH: Data curation, Formal analysis, Software, Visualization, Writing – original draft. YZ: Data curation, Formal analysis, Project administration, Software, Visualization, Writing – original draft. QZ: Conceptualization, Methodology, Project administration, Resources, Supervision, Validation, Writing – review & editing. LK: Conceptualization, Investigation, Methodology, Project administration, Resources, Supervision, Validation, Writing – original draft, Writing – review & editing.
