# Supplementary material for: Effectiveness of resistance training in modulating inflammatory biomarkers among Asian patients with sarcopenia: a systematic review and meta-analysis of randomized controlled trials
Source: Front Immunol. 2024 May 28;15:1385902. doi: 10.3389/fimmu.2024.1385902 (PMC11165069; doi:10.3389/fimmu.2024.1385902)
Supplement: Supplementary file 1 [file DataSheet_1.docx]

**Supplementary Table 1.**

**Search strategy for each database**

1. **PubMed**

((Inflammation[Title/Abstract]) OR (Innate Inflammatory Response[Title/Abstract]) OR (C Reactive Protein[Title/Abstract]) OR (hs-CRP[Title/Abstract]) OR (Interleukin 6[Title/Abstract]) OR (IL6[Title/Abstract]) OR (Interleukin 10[Title/Abstract]) OR (IL10[Title/Abstract]) OR (Tumor Necrosis Factor alpha[Title/Abstract]) OR (TNF alpha[Title/Abstract]) OR (Inflammation[Mesh])) AND ((Resistance Training[Title/Abstract]) OR (Resistance Training[Title/Abstract]) OR (Resistance Exercise[Title/Abstract]) OR (Strength Training[Title/Abstract]) OR (Strength Exercise[Title/Abstract]) OR (Weight Lifting Strengthening Program[Title/Abstract]) OR (Weight Lifting Exercise Program[Title/Abstract]) OR (Weight Bearing Strengthening Program[Title/Abstract]) OR (Weight Bearing Exercise Program[Title/Abstract]) OR (Resistance Training[Mesh])) AND ((Sarcopenia[Title/Abstract]) OR (Sarcopenias[Title/Abstract]) OR (Sarcopenia[Mesh]))

**111 results**

1. **Cochrane library**

#1 (Sarcopenias OR Sarcopenia):ti,ab,kw

#2 (Inflammation OR Innate Inflammatory Response OR C Reactive Protein OR hs-CRP OR Interleukin 6 OR IL6 OR Interleukin 10 OR IL10 OR Tumor Necrosis Factor alpha OR TNF alpha):ti,ab,kw

#3 (Resistance Training OR Resistance Exercise OR Strength Training OR Strength Exercise OR Weight Lifting Strengthening Program OR Weight Lifting Exercise Program OR Weight Lifting Exercise Program OR Weight Lifting Exercise Program OR Weight Bearing Strengthening Program OR Weight Bearing Exercise Program):ti,ab,kw

#1 AND #2 AND #3

**113 results**

1. **Embase**

#1 inflammation:ab,ti OR innate inflammatory response:ab,ti OR c reactive protein:ab,ti OR hs crp:ab,ti OR interleukin 6:ab,ti OR il 6:ab,ti OR interleukin 10:ab,ti OR il10:ab,ti OR tumor necrosis factor alpha:ab,ti OR tnf alpha:ab,ti

#2 resistance training:ab,ti OR resistance exercise:ab,ti OR strength exercise:ab,ti OR weight lifting strengthening program:ab,ti OR weight lifting exercise program:ab,ti OR weight bearing strengthening program:ab,ti OR weight bearing exercise program:ab,ti

#3 sarcopenia:ab,ti OR sarcopenias:ab,ti

#1 AND #2 AND #3

**84 results**

1. **Web of science**

#1 TS=(Sarcopenia)) OR TS=( Sarcopenias)

#2 TS=(Inflammation) OR TS=(Innate Inflammatory Response) OR TS=(C Reactive Protein) OR TS=( hs-CRP) OR TS=(Interleukin 6) OR TS=(IL6) OR TS=(Interleukin 10) OR TS=(IL10) OR TS=( Tumor Necrosis Factor alpha) OR TS=(TNF alpha)

#3 TS=(Resistance Training) OR TS=(Resistance Exercise) OR TS=(Strength Training) OR TS=( Strength Exercise) OR TS=(Weight Lifting Strengthening Program) OR TS=(Weight Lifting Exercise Program) OR TS=(Weight Lifting Exercise Program) OR TS=(Weight Lifting Exercise Program) OR TS=(Weight Bearing Strengthening Program) OR TS=(Weight Bearing Exercise Program)

#1 AND #2 AND #3

**630 results**

**Total 938 results**

**Supplementary figure 1. Publication bias was performed on the effects of resistance training on IL-6 (A), TNF-α (B), and CRP (C) in sarcopenia.**

**
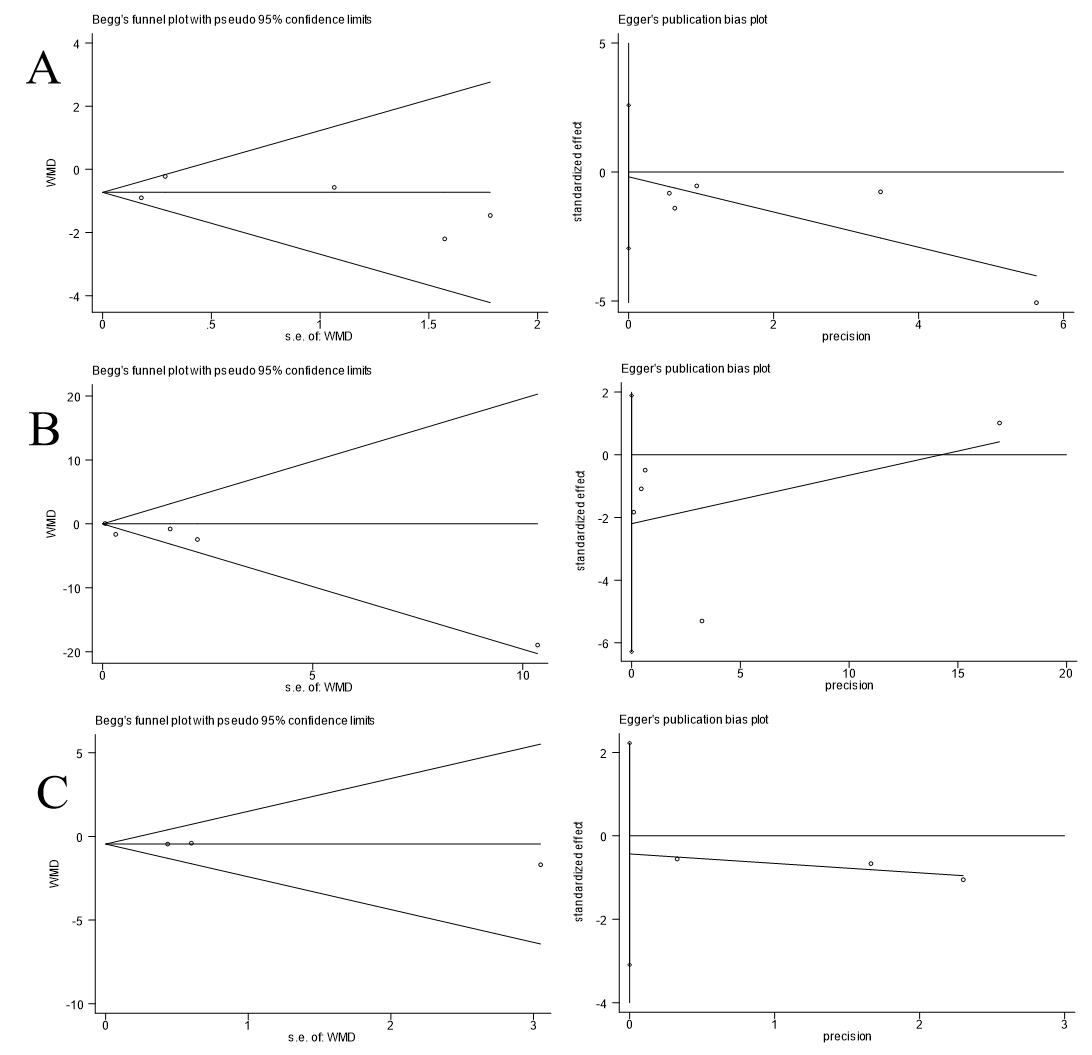
**

**Supplementary Table 2. GRADE Approach for certainty of evidence.**

| **Certainty assessment** | | | | | | | **№ of patients** | | **Effect** | | **Certainty** |
| --- | --- | --- | --- | --- | --- | --- | --- | --- | --- | --- | --- |
| **№ of studies** | **Study design** | **Risk of**  **bias** | **Inconsistency** | **Indirectness** | **Imprecision** | **Other**  **considerations** | **RT** | **CG** | **Relative**  **(95% CI)** | **Absolute**  **(95% CI)** |  |
| **IL-6** | | | | | | | | | | | |
| 5 | RCTs | serious^a^ | not serious | not serious | not serious | none | 120 | 119 | - | WMD **0.73 lower** (1.02 lower to  0.44 lower) | ⨁⨁⨁◯  Moderate |
| **TNF-α** | | | | | | | | | | | |
| 5 | RCTs | serious^b^ | not serious^c^ | not serious | serious^d^ | none | 111 | 99 | - | WMD **1**  **lower** (2.47 lower to  0.46 higher) | ⨁⨁◯◯  Low |
| **CRP** | | | | | | | | | | | |
| 3 | RCTs | serious^e^ | not serious | not serious | serious^d^ | none | 76 | 64 | - | WMD **0.45 lower** (1.14 lower to  0.24 higher) | ⨁⨁◯◯  Low |
| **IL-10** | | | | | | | | | | | |
| 2 | RCTs | serious^f^ | seriousg | not serious | serious^d^ | none | 41 | 40 | - | WMD **0.13 higher** (3.99 lower to  4.25 higher) | ⨁◯◯◯ Very low |

**RT:** resistance training; **CG:** control group; **RCTs:** randomized controlled trials; **CI:** confidence interval; **WMD:** weighted mean difference.

**Explanations**

a. The quality for 5 of 5 trials was unclear. The main trial limitation was the lack of random sequence generation, allocation concealment, and double-blinding. Downgraded.

b. The quality for 5 of 5 trials was unclear. The main trial limitation was the lack of random sequence generation, allocation concealment, and double-blinding. Downgraded.

c. I^2^ = 88.2%, P < 0.001. This study subgroup explained the observed heterogeneity. [Mixed-gender: WMD = -1.64, 95% CI = -2.60 to -0.46, P = 0.001, I^2^ = 6.3%; Frequency at least three times per week: WMD = -1.65, 95% CI = -2.26 to -1.05, P < 0.001, I^2^ = 0.0%; Period at least 12 weeks: WMD = -1.64, 95% CI = -2.60 to -0.46, P = 0.001, I^2^ = 6.3%]. Not downgraded.

d. 95% CI crosses the threshold of zero.

e. The quality for 4 of 4 trials was unclear. The main trial limitation was the lack of random sequence generation, allocation concealment, and double-blinding. Downgraded.

f. The quality for 2 of 2 trials was unclear. The main trial limitation was the lack of random sequence generation, allocation concealment, and double-blinding. Downgraded.

g. I^2^ = 80.6, *P* < 0.001. The predefined subgroups and sensitivity analyses did not explain the source of heterogeneity. Downgraded.
